# Dose–response effects of dietary inclusion of agro‐industrial by‐products on *in vitro* ruminal fermentation and methane production

**DOI:** 10.1002/jsfa.14263

**Published:** 2025-04-08

**Authors:** Benchu Xue, Joshua P. Thompson, Tianhai Yan, Sokratis Stergiadis, Laurence Smith, Katerina Theodoridou

**Affiliations:** ^1^ Institute for Global Food Security Queen's University Belfast Belfast UK; ^2^ Sustainable Livestock Systems Agri‐Food and Biosciences Institute Hillsborough UK; ^3^ School of Agriculture, Policy and Development University of Reading Reading UK

**Keywords:** food by‐products, ruminants, feed ingredients, *in vitro* fermentation, methane emission

## Abstract

**BACKGROUND:**

As the agro‐industry produces considerable amounts of by‐products globally, it is acknowledged that there is a need to address the environmental issues related to their disposal and the resource competition between food for humans and feed for animals. The aim of this study was to explore, *in vitro*, the effects of various by‐products from the agro‐industry on rumen fermentation and methane emission. Samples were collected from various food processing industries, including red and green apple pomace (RAP, GAP), hempseed cake (HC), coffee hulls (CH), coffee grounds (CG), spent mushroom compost (SMC) and distiller's dried grains with solubles (DDGS). In doses of 100, 200 and 300 g kg^−1^, the tested by‐products were incubated in rumen fluid, where the by‐products replaced equal amounts of substrates.

**RESULTS:**

Gas production (GP) and dry matter digestibility (DMD) decreased linearly for most of the tested by‐products with the growth of doses (*P* < 0.001), while NH_3_‐N concentration increased linearly. Linear decreases were observed in CH_4_ production with increasing doses of all by‐products (*P* < 0.05). The reduction of CH_4_ production ranged from 21.4% to 33.6% at doses of 100–300 g kg^−1^, but reductions were only observed at a dose of 100 g kg^−1^ when CH_4_ productions were corrected by digested dry matter (*P* < 0.05). RAP, GAP and HC were higher than CH, CG and SMC for the comparison of key parameters including DMD, GP and volatile fatty acids. Better methane‐mitigating effects were observed for RAP, GAP and HC than for the control group and CH, CG and SMC.

**CONCLUSION:**

Most of the by‐products tested were found to be a potential option for replacing conventional feed ingredients but should not exceed a dose at 200 g kg^−1^. © 2025 The Author(s). *Journal of the Science of Food and Agriculture* published by John Wiley & Sons Ltd on behalf of Society of Chemical Industry.

## INTRODUCTION

The total amount of agro‐industrial by‐products in the UK is 2.6 million tons, which makes the UK one of the top producers in Europe, only second to Germany with 3 million tons.^1^ Agro‐industrial by‐products are residual materials generated during the processing of agricultural and industrial products. These by‐products often retain substantial nutritional or varieties of bioactive components, which depend on the type of by‐products itself and the processing methods.^2^, ^3^ Nevertheless, nearly half of those by‐products are disposed of in unsustainable procedures such as landfilling and incineration, causing considerable negative impact on the environment.^4^ Characterized by high fibre and protein content, by‐products from the food processing industry are widely used for animal feed, especially ruminants, as fibre can be the primary source of their energy supply while not digestible for monogastric livestock. Industry and academia all over the world are increasingly focusing on sustainable approaches to minimize waste and extract value from these by‐products, in which transforming them to animal feed is of relatively high efficiency.^5^

As previous studies reported, the doses of by‐products were found to be of vital importance when included in diets for livestock.^6^ Researchers have summarized the safe doses at which fruit by‐products can be added to the diets of different livestock, where the feeding risk has been taken into prime consideration.^7^ However, doses need to be more accurate when it comes to large‐scale farms, so it is greatly necessary to conduct *in vitro* assessments for these by‐products to clarify the dose–response effects, thereby providing feasibility and guidance on *in vivo* experiments and their application. A study investigated the *in vitro* effect of citrus pulp inclusion levels at 0, 100, 200 and 300 g kg^−1^ dry matter (DM) in total mixed ration (TMR) in goats. The digestibility of organic matter and DM increased linearly with the inclusion levels, while short‐chain fatty acids and metabolizable energy reached a maximum at 300 g kg^−1^.^8^ Another study that conducted *in vitro* trials by replacing dried tomato pomace in diets at doses of 60, 120 and 180 g kg^−1^ DM found differences in total volatile fatty acids (VFA) and NH_3_‐N after 8 h incubation among groups, but most of the difference disappeared after 24 h.^9^ Furthermore, species‐specific differences in optimal doses are significant in *in vivo* study, even for the same by‐products. A study that set coffee grounds inclusion levels at 0, 100 and 200 g kg^−1^ DM in TMR found that increasing levels linearly decreased the digestibility of DM, protein, fibre and retained nitrogen.^10^ The DMI decreased to the lowest when feeding at 200 g kg^−1^ DM dose (96.6 *versus* 94.8 *versus* 76.8 g/(body weight)^0.75^). Intriguingly, two research studies on dairy cows, that also set 100 and 200 g kg^−1^ DM inclusion levels, respectively, found no significant difference in the VFA, N‐nitrogen and gas production among groups.^11^, ^12^

*In vitro* studies related to agro‐industrial by‐products usually focus on rumen fermentation parameters,^13^ but ignore the presence of bioactive compounds, such as total phenolics, which have already proved to be of high efficiency in inhibiting rumen methanogenic archaea.^14^, ^15^ For specific by‐products rich in phenolics, the mitigation of CH_4_ production in *in vitro* studies is rarely reported. More importantly. although a great number of potential by‐products and their potentially optimal doses were explored for *in vitro* incubation,^16^, ^17^ more accurate information is poorly investigated when it comes to the comparison among the by‐products from different agro‐industries. In the study reported here, samples were collected from various food processing industries, including juice, coffee, whiskey, mushroom and hempseed. The different effects caused by dose and type of by‐product are equally important for us to focus on, allowing selection of the best by‐product candidates for application in animal trials.

Therefore, the aim of the study was to explore, *in vitro*, the effects of various by‐products from agro‐industry on rumen fermentation and methane emission. The specific objectives were: (1) to explore the nutritive value and total phenolics of the by‐products collected and (2) to assess the dose–response effects of including increasing doses (100, 200, 300 g kg^−1^ DM) of the seven by‐products on *in vitro* ruminal fermentation and methane production.

## MATERIALS AND METHODS

### Preparation of samples

The agro‐industrial by‐products used in this study were red and green apple pomace (RAP, GAP), hempseed cake (HC), coffee hulls (CH), coffee grounds (CG), spent mushroom compost (SMC) and distiller's dried grains with solubles (DDGS). RAP and GAP were obtained from MacNeice Fruit Ltd and Moorstown, Co. Tipperary, Ireland, respectively. HC was collected from UK Hemp Co., UK. CH were collected from a local micro‐roastery based in Belfast, UK. CG were collected from a local coffee shop in Belfast. SMC was from Agri‐Food and Bioscience Institute (AFBI). The rest of the substrates for *in vitro* incubation, silage and concentrate were collected from AFBI in Hillsborough, Northern Ireland, UK. All samples were dried in a freeze dryer for 72 h, and then ground to pass through a 1 mm sieve.

### Experimental design

Experiments were conducted to measure *in vitro* rumen fermentation characteristics of diets with different inclusion levels of agro‐industrial by‐products, to determine the dose–response effects. Tested by‐products on DM basis were incubated for 24 h in doses of 100, 200 and 300 g kg^−1^ DM substrates. These amounts replaced equal amounts of the mixed ration (500 mg), which was composed of silage and concentrate (70:30).

Based on a literature review, those by‐products with low protein content, apple pomace, coffee products and mushroom compost, were designed to replace the silage portion, while those with high protein content, HC and DDGS, were designed to replace the concentrate portion, keeping nitrogen balanced in the diets. Details are presented in Table 1. Each of the 21 treatments (7 by‐products × 3 doses) was repeated in two independent *in vitro* runs over 2 weeks. In addition, each run included triplicate of treatment diets, quadruplicate of control diets (substrate alone without by‐products) and blanks. The total number of experimental units were (21 treatments × 3 + control diets × 4) × 2 runs = 134, which were used in the statistical analysis.

**Table 1:** Composition of individual groups for *in vitro* experiments

	Inclusion level (g kg^−1^)	Silage (g kg^−1^)	Concentrate (g kg^−1^)	By‐products (g kg^−1^)
Replacing silage^a^	100	600	300	100
	200	500	300	200
	300	400	300	300
Replacing concentrate^b^	100	700	200	100
	200	700	100	200
	300	700	0	300

^a^ By‐products include red apple pomace, green apple pomace, coffee hull, coffee grounds and spent mushroom compost.

^b^ By‐products include hempseed cake and distiller's dried grains with solubles.

### Experimental procedures and sampling

*In vitro* incubations for this study were performed according to Menke and Steingass.^18^ Rumen fluid was collected before the morning feeding from three cannulated non‐lactating Holstein cows fed a ration consisting of 700 g kg^−1^ grass silage and 300 g kg^−1^ of a commercial concentrate mix (133 ± 7.8 g kg^−1^ crude protein (CP), 33 ± 5.8 g kg^−1^ ether extract (EE), 275 ± 6.1 g kg^−1^ neutral detergent fibre (NDF) and 99 ± 1.1 g kg^−1^ ash) twice daily at the abattoir centre for AFBI (1 L from each cow). The rumen contents were transferred into three thermos flasks and immediately transported to the laboratory. Rumen contents were strained through four layers of cheesecloth into an Erlenmeyer flask, followed by mixing with 2× *in vitro* rumen buffer solution under strictly anaerobic conditions.^19^ Subsequently, the buffered rumen fluids from three cattle were homogenized. An amount of 50 mL of rumen fluid/buffer mixture with a ratio of 1:2 was transferred into 125 mL serum bottles containing 0.5 g of experimental diets prepared already under continuous flushing with O_2_‐free CO_2_ gas. The bottles were sealed with butyl rubber stoppers and caps made of aluminium and incubated at 39 °C with continuous rotation for 24 h.

At the end of incubation, for each serum bottle, gas production was measured using a pressure transducer and a syringe to collect gas stored for 24 h in a 20 mL gas chromatography vial for methane (CH_4_) analysis. Once opened, the entire contents of each serum bottle were transferred to a pre‐weighed 50 mL falcon tube, followed by measuring pH immediately. Supernatants were sampled into 2 mL Eppendorf tubes frozen at −20 °C for VFA and NH_3_‐N analysis. Each serum bottle was washed twice with distilled water to recover all the nondegraded particles that were transferred into the 50 mL falcon tube. Tubes were centrifuged at 3400 rpm for 10 min at 4 °C. Once the supernatant was removed, the residue was obtained, followed by transferring to an oven immediately.

### Chemical analysis

For the chemical composition of by‐products, contents of ash (method 942.05) and EE (method 920.39) were analysed as described by AOAC (2000).^20^ Concentrations of NDF and acid detergent fibre (ADF) were determined following the procedures of Van Soest *et al*.^21^ and Robertson and Van Soest,^22^ respectively, using an ANKOM220 fibre analyser unit (ANKOM Technology Corporation, Fairport, NY, USA). Nitrogen (N) concentrations were determined by the Dumas combustion technique employing a Leco FP258 N analyser (Leco Corporation, St Joseph, MI, USA), and CP concentration (g kg^−1^ DM) was then calculated as N concentration × 6.25. Concentrations of NH_3_‐N were determined by the phenol–hypochlorite method.^23^ The concentration of VFA was analysed using gas chromatography as described by Huhtanen *et al*.^24^ Non‐fibrous carbohydrate (NFC) was calculated as 1000 – CP − EE – ash − NDF based on NRC (2001).^25^ All analyses were performed in triplicate. Gas production (GP) was calculated based on pressure measurements according to the following equation^26^:

$$\mathrm{GP}=\frac{V_{h}}{P_{a}}\times P_{t}$$

where *V*_h_ represents head‐space volume (mL), *P*_a_ atmospheric pressure (psi) and *P*_t_ pressure transducer reading (psi). Standard *P*_a_ value of 14.7 psi was used and *V*_h_ value of 70 mL.

### Quantification of total phenolics in by‐products

Measurement of total phenolics was conducted based on the methods using Folin–Ciocalteu reagent.^27^ Total phenolics were extracted from the seven by‐products in triplicate through 70% aqueous acetone (*n* = 3), followed by a series of different dilutions, ×2, ×5, ×20, mixed with Folin–Ciocalteu reagent and Na_2_CO_3_ reagent, and finally absorbance measurements of each sample were recorded at 725 nm using a spectrophotometer.

### Measurement of methane production

Gas samples were collected from bottles’ headspace for methane (CH₄) analysis. A Terumo™ Agani™ 18‐gauge, 1.5‐inch needle, coupled with a gas stopper and a 12 mL syringe, was employed to extract 10 mL of gas from each serum bottle, which was then transferred into a 12 mL evacuated Exetainer® vial. Methane concentration was quantified via gas chromatography using an HP 5890 Series II chromatograph with an HP‐Innovax column (25 mm × 0.2 mm × 0.2 μm, Supelco). The carrier gas was nitrogen at 1 mL min^−1^. The injector and detector temperatures were maintained at 250 and 275 °C, respectively, while the oven temperature was held at 110 °C under isothermal conditions. A 0.1 mL gas sample was injected with a 1 mL sample‐lock syringe. Methane levels were calibrated using a standard curve created through manual injections of six different quantities of pure CH₄ in triplicate, and the final CH₄ concentration was expressed in mL of CH₄ per mL of sample.

### Calculation and statistics analysis

Calculation of the *in vitro* DM digestibility (IVDMD) was conducted as follows:

$$\text{IVDMD}\;\left(\%\right)=\left[\left(X-Y\right)/X\right]\times 100$$

where *X* = initial weight (g) and *Y* = dry residue weight (g).

Calculation of total phenolics contents (for use with ×20 dilution) was as follows:

$$\text{Total phenolics}\;\left(\%\right)=X/Y\times 10$$

where *X* = phenolics (μg) and *Y* = dry residue weight (mg). Results were expressed as mg gallic acid equivalent (GAE) per g DM.

All data were analysed using mixed linear model in SPSS (Ver.22.0 for Windows; SPSS, Chicago, IL, USA). Model 1 was used to evaluate the overall effects of by‐products on rumen fermentation, with by‐products, dose and their interaction as fixed effects, run as random effect. Model 2 was used to compare the by‐products' effects within the doses and doses’ effects within the by‐products. Linear and quadratic effects of dose within by‐products were evaluated by orthogonal polynomial contrasts. *Post hoc* multiple comparisons were performed using the Sidak test. These results are presented as means and standard error of means. Statistical differences were declared significant when *P* < 0.05 and declared as a tendency when 0.05 ≤ *P* < 0.10.

## RESULTS

### Chemical composition of ingredients and experimental diets

The CP content was highest in HC at 376.9 g kg^−1^ DM, numerically followed by DDGS at 317 g kg^−1^ DM, while for the others it was below 170 g kg^−1^ (Table 2). The highest NDF was found in CH and CG, over 630 g kg^−1^ DM, numerically followed by SMC and HC, whereas RAP and GAP had the lowest at 346 and 318 g kg^−1^. The ADF content of each by‐product was numerically lower than the NDF content (210–529 g kg^−1^ DM). Apple by‐products have the highest of NFC at 670 g kg^−1^ DM, while coffee by‐products and SMC have the lowest. Fat and ash contents range from 29 to 136 g kg^−1^ DM and from 18 to 230 g kg^−1^ DM respectively.

**Table 2:** Chemical composition of substrate and tested by‐products

Item	Diet ingredients								
	GS	Con	RAP	GAP	HC	CH	CG	SMC	DDGS
DM (g kg^−1^)	918	942	923	895	899	947	983	945	889
GE (MJ kg^−1^ DM)	19.2	18.1	13.4	18.4	22.0	20.5	23.0	16.2	21.6
CP (g kg^−1^ DM)	137	270	53.0	65.0	327	165	155	145	317
EE (g kg^−1^ DM)	48.0	27.0	29.0	37.0	88.0	15.0	136	31.0	60.0
NDF (g kg^−1^ DM)	441	285	346	318	359	530	537	552	370
ADF (g kg^−1^ DM)	297	182	210	210	309	429	400	426	225
Ash (g kg^−1^ DM)	11.0	76.0	18.0	20.0	77.0	70.0	21.0	230	59.0
NFC (g kg^−1^ DM)	363	342	554	560	150	221	151	43.0	194

DM, dry matter; GE, gross energy; CP, crude protein; EE, ether extract; NDF, neutral detergent fibre; ADF, acid detergent fibre; NFC, non‐fibrous carbohydrate; GS, grass silage; Con, concentrate, RAP, red apple pomace; GAP, green apple pomace; HC, hempseed cake; CH, coffee hull; CG, coffee grounds; SMC, spent mushroom compost; DDGS, distiller's dried grain and solubles.

For chemical composition in each dietary treatment (Table 3), CP ranged from 152 to 194 g kg^−1^ DM, with HC and DDGS diets having the highest. NDF ranged from 366 to 428 g kg^−1^ DM and ADF ranged 236 to 302 g kg^−1^ DM, with SMC diets having the highest content. A numerically noticeable difference is the ash content in SMC diets at 52.4–92.2 g kg^−1^ DM but there was only 30.5 g kg^−1^ DM in control diets. Fat contents in HC and CG diets at 47.8–60 and 50.5–68.1 g kg^−1^ DM, respectively, were numerically higher than that of control at 41.7 g kg^−1^ DM. NFC ranged from 261 to 416 g kg^−1^ DM, with RAP and GAP diets having the highest.

**Table 3:** Chemical composition of experimental diets

Item		Dietary treatments																				
	CON	RAP 10	RAP 20	RAP 30	GAP 10	GAP 20	GAP 30	HC 10	HC 20	HC 30	CH 10	CH 20	CH 30	CG 10	CG 20	CG 30	SMC 10	SMC 20	SMC 30	DDGs 10	DDGs 20	DDGs 30
Chemical composition (g kg^−1^)																						
DM	925	926	926	927	923	921	918	926	926	927	928	931	934	932	938	945	928	931	933	920	915	909
OM	970	969	968	967	969	968	967	971	972	973	964	958	952	969	968	967	948	926	904	971	973	975
GE	18.9	18.3	17.7	17.1	18.8	18.7	18.6	19.3	19.7	20.0	19	19.1	19.3	19.3	19.6	20.0	18.6	18.3	18.0	19.2	19.6	19.9
CP	178	169	160	152	170	163	155	183	188	194	180	183	185	179	181	182	178	179	179	182	186	191
NDF	394	385	375	366	382	370	357	402	409	416	403	412	422	404	413	423	405	416	428	403	411	420
ADF	263	254	245	236	254	245	236	275	288	301	276	289	302	273	283	293	275	288	301	267	271	275
EE	41.7	39.8	37.9	36	40.6	39.5	38.4	47.8	53.9	60	38.0	35.0	32.0	50.5	59.3	68.1	40	38.3	36.6	45.0	48.3	51.6
Ash	30.5	31.2	31.9	32.6	31.4	32.3	33.2	29.5	28.5	27.5	36.4	42.3	48.2	31.5	32.5	33.5	52.4	74.3	96.2	28.8	27.1	25.4
NFC	357	376	395	414	376	396	416	338	318	299	343	330	316	336	314	293	325	293	261	342	327	312

DM, dry matter; OM, organic matter; GE, gross energy; CP, crude protein; NDF, neutral detergent fibre; ADF, acid detergent fibre; EE, ether extract; NFC, non‐fibrous carbohydrate; CON, control diet; RAP, red apple pomace; GAP, green apple pomace; HC, hempseed cake; CH, coffee hull; CG, coffee grounds; SMC, spent mushroom compost; DDGS, distiller's dried grain and solubles; 10, 20 and 30, inclusion level of each by‐product at 10%, 20% and 30%.

### Total phenolics contents of by‐products

**Figure 1:**
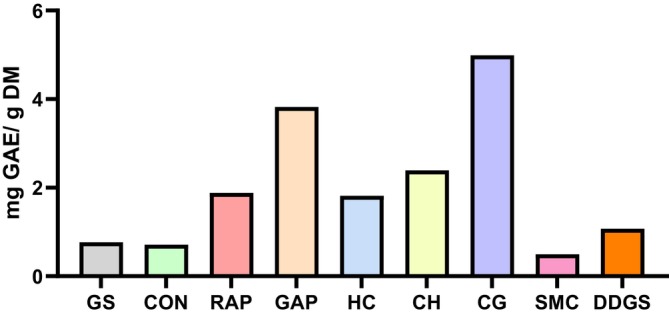
Analysed total phenolics (mg GAE g^−1^ DM) of experimental substrates. GAE, gallic acid equivalent; GS, grass silage; CON, concentrate; RAP, red apple pomace; GAP, green apple pomace; HP, hempseed cake; CH, coffee hull; CG, coffee grounds; SMC, spent mushroom compost; DDGS, distiller's dried grains with solubles.

The total phenolics contents contained in the tested by‐products are presented in Fig. 1. The two highest levels of total phenolics contents were found in CG and GAP, at 4.99 and 3.82 mg GAE g^−1^ DM, followed by those of CH, RAP, HC and DDGS, at 2.39, 1.88, 1.82 and 1.07 mg GAE g^−1^ DM. SMC had the lowest level of total phenolic content at 0.5 mg GAE g^−1^ DM.

### Fermentation characteristics

Based on model 1 (Table 4), IVDMD and pH were significantly affected by the doses and by‐products (*P* < 0.001) and significant interactions were observed (*P* < 0.001). Based on model 2, the pH linearly increased with increasing doses of HC, CH, CG and SMC (*P* < 0.001). As the dose of by‐products increased, a linear decline in IVDMD was observed for most of the by‐products tested (*P* < 0.001), except RAP with a quadratic trend. CH, CG and SMC at each dose was lower than other groups (*P* < 0.05). At doses of 100 g kg^−1^ DM, lower IVDMD and GP were observed for CH, CG and SMC while for doses of 20 and 300 g kg^−1^ DM, the differences were more significant (*P* < 0.05).

**Table 4:** IVDMD and pH after 24 h *in vitro* incubation of the experimental diets with different inclusion levels of various by‐products using strained ruminal fluid

Item	Dose (g kg^−1^)	CON	RAP	GAP	HC	CH	CG	SMC	DDGs	SE	*P*		
											Type	Dose	T × D
IVDMD (%)										0.63	<0.001	<0.001	<0.001
	100	65.0^a^	66.0^a^	64.9^a^	62.2^ab^	60.1^b^	59.2^b^	60.5^b^	62.5^ab^				
	200	65.0^ab^	68.7^a^	63.6^abc^	49.4^e^	56.5^cde^	56.3^de^	58.3^bcd^	62.3^abcd^				
	300	65.0^a^	64.7^a^	57.7^abc^	46.2^e^	53.1^cde^	48.5^de^	54.7^bcd^	62.0^ab^				
	Contrast^†^		L	L	L	L	L	L	L				
pH										0.01	<0.001	<0.001	<0.001
	100	6.18^c^	6.20^bc^	6.19^c^	6.21^abc^	6.24^abc^	6.26^ab^	6.27^a^	6.18^c^				
	200	6.18^bc^	6.22^b^	6.15^c^	6.29^a^	6.28^a^	6.28^a^	6.31^a^	6.16^bc^				
	300	6.18^b^	6.18^b^	6.14^b^	6.33^a^	6.32^a^	6.33^a^	6.34^a^	6.19^b^				
	Contrast		Q	L	L	L	L	L Q	—				

^a, b, c, d, e^ Significant differences between by‐products are indicated with different superscript letters (*P* < 0.05).

IVDMD, *in vitro* dry matter digestibility; CON, control diets; RAP, red apple pomace; GAP, green apple pomace; HP, hempseed cake; CH, coffee hull; CG, coffee grounds; SMC, spent mushroom compost; DDGS, distiller's dried grains with solubles; SE, standard error of mean; T × D, type × dose.

^†^ Significant (*P* < 0.05) linear (L) or quadratic (Q) contrasts of the response to incremental doses (from 0 to 300 g kg^−1^) of each by‐product.

**Figure 2:**
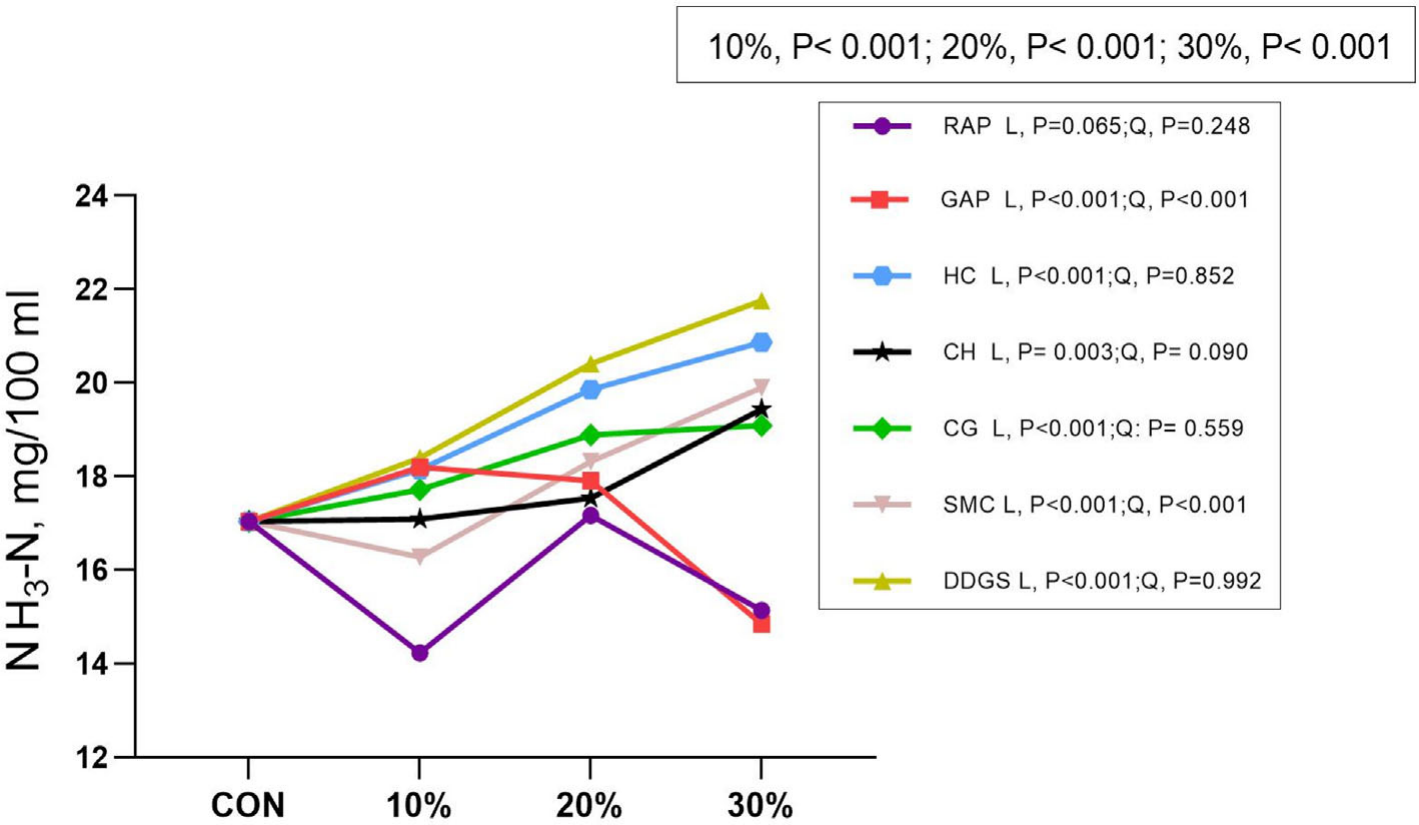
Effect of dietary inclusion of agro‐industrial by‐products on ruminal *in vitro* ammonia concentration. RAP, red apple pomace; GAP, green apple pomace; HP, hempseed cake; CH, coffee hull; CG, coffee grounds; SMC, spent mushroom compost; DDGS, distiller's dried grains with solubles. 10%, comparison among by‐products at the dose of 10%; 20%, comparison among by‐products at the dose of 20%; 30%, comparison among by‐products at the dose of 30%; L and Q, linear and quadratic of orthogonal polynomial contrasts of the response to incremental doses (from 0% to 30%) of by‐products.

**Figure 3:**
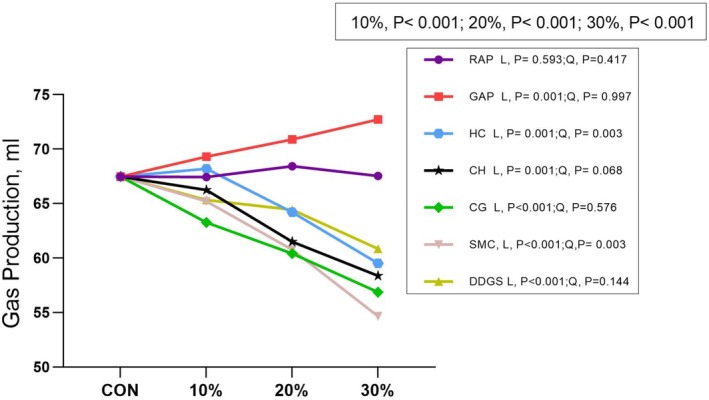
Effect of dietary inclusion of agro‐industrial by‐products on ruminal *in vitro* gas production. RAP, red apple pomace; GAP, green apple pomace; HP, hempseed cake; CH, coffee hull; CG, coffee grounds; SMC, spent mushroom compost; DDGS, distiller's dried grains with solubles. 10%, comparison among by‐products at the dose of 10%; 20%, comparison among by‐products at the dose of 20%; 30%, comparison among by‐products at the dose of 30%; L and Q, linear and quadratic of orthogonal polynomial contrasts of the response to incremental doses (from 0% to 30%) of by‐products.

Based on model 1 (Figs 2 and 3), NH_3_‐N and GP were significantly affected by the doses and by‐products (*P* < 0.001) and significant interactions were observed (*P* < 0.001). Based on model 2, most of the tested by‐products linearly decreased NH_3_‐N and GP (*P* < 0.05).

### Production of volatile fatty acids

Based on model 1 (Table 5), by‐product type caused significant effects on the production of VFA (*P* < 0.001) but doses did not. Based on model 2, inclusion of HC, CH, CG and SMC linearly decreased total VFA (*P* < 0.05). Similar trends were observed in the production of individual VFA, including acetate, propionate, butyrate and valerate (*P* < 0.05). With an increase in the dose of by‐products, the acetate‐to‐propionate ratio (A:P) linearly increased for CH, CG and SMC (*P* < 0.05), while linear decreases were observed for RAP, GAP and DDGS (*P* < 0.05). At doses of 100, 200 and 300 g kg^−1^ DM of by‐product inclusion, all individual VFA were lower in CG and SMC than in other groups (*P* < 0.05). At all doses of by‐product inclusion, A:P of HC, CH, CG and SMC was significantly higher than that of RAP, GAP and DDGS (*P* < 0.05).

**Table 5:** Total volatile fatty acids, acetate, propionate, butyrate, valerate production and ratio of acetate and propionate after 24 h *in vitro* incubation of the experimental diets with different inclusion levels of various by‐products using strained ruminal fluid

Item	Dose (g kg^−1^)	CON	RAP	GAP	HC	CH	CG	SMC	DDGS	SE	*P*		
											Type	Dose	T × D
TVFA (mmol L^−1^)										1.32	<0.001	0.325	0.053
	100	91.2^a^	85.2^a^	91.3^a^	89.9^a^	88.5^a^	81.6^ab^	64.2^b^	90.5^a^				
	200	91.2^a^	84.8^a^	87.7^a^	84.3^a^	88.4^a^	62.5^b^	79.0^ab^	94.7^a^				
	300	91.2^a^	88.7^a^	88.8^a^	85.2^a^	79.6^a^	58.3^b^	75.5^ab^	88.1^a^				
	Contrast^†^		—	—	L	L	L	Q	—				
Acetate (mmol L^−1^)										0.93	<0.001	0.308	0.081
	100	60^a^	55.5^ab^	60.0^a^	59.6^a^	59.0^a^	51.2^ab^	43.7^b^	59.8^a^				
	200	60^a^	54.7^ab^	56.3^a^	55.1^a^	59.8^a^	42.8^b^	54.0^ab^	62.2^a^				
	300	60^a^	57.4^a^	56.4^a^	54.7^a^	54.5^a^	40.3^b^	52.2^ab^	57.6^a^				
	Contrast		—	L	L	—	L	Q	—				
Propionate										0.42	<0.001	0.465	0.015
(mmol L^−1^)	100	21.4^a^	21.5^a^	22.1	19.8^a^	19.8^a^	17.9^ab^	13.1^b^	21.3^ab^				
	200	21.4^a^	21.6^a^	22.0^a^	19.5^ab^	18.9^ab^	12.7^c^	16.4^bc^	22.8^a^				
	300	21.4^ab^	22.9^a^	22.1^a^	20.1^abc^	16.5^bc^	11.2^d^	15.1^cd^	22.2^a^				
	Contrast		—	—	—	L	L	L Q	—				
Butyrate										0.20	<0.001	0.331	0.07
(mmol L^−1^)	100	7.31^ab^	6.13^ab^	6.94^ab^	7.66^a^	7.28^ab^	6.66^ab^	5.48^b^	6.89^ab^				
	200	7.31^a^	6.36^ab^	7.09^a^	6.85^ab^	7.06^a^	5.30^b^	6.39^ab^	7.02^a^				
	300	7.31^ab^	6.39^abc^	7.91^a^	7.17^ab^	6.32^abc^	5.03^c^	5.98^abc^	5.88^bc^				
	Contrast		—	—	—	L	L	L Q	L				
Valerate										0.02	<0.001	0.312	0.016
(mmol L^−1^)	100	0.95^ab^	0.82^bc^	0.93^ab^	1.06^a^	0.95^ab^	0.85^abc^	0.70^c^	1.01^ab^				
	200	0.95^ab^	0.80^bc^	0.87^ab^	1.04^a^	0.91^ab^	0.65^c^	0.77^bc^	1.05^a^				
	300	0.95^ab^	0.83^bc^	0.96^ab^	1.19^a^	0.78^bcd^	0.59^d^	0.70^cd^	0.98^ab^				
	Contrast		L Q	—	L	L	L	L Q	—				
A:P										0.06	<0.001	0.648	<0.001
	100	2.91^abc^	2.72^c^	2.83^bc^	3.10^ab^	3.09^ab^	2.98^abc^	3.20^a^	2.88^bc^				
	200	2.91^c^	2.57^d^	2.70^cd^	2.98^bc^	3.29^a^	3.26^ab^	3.33^a^	2.78^cd^				
	300	2.91^b^	2.56^c^	2.71^ab^	2.78^ab^	3.41^a^	3.40^a^	3.51^a^	2.66^ab^				
	Contrast		L	L	Q	L	L	L	L				

^a, b, c, d^ Significant differences between by‐products are indicated with different superscript letters (*P* < 0.05).

TVFA, total volatile fatty acids; A:P, acetate:propionate; CON, control diets; RAP, red apple pomace; GAP, green apple pomace; HP, hempseed cake; CH, coffee hull; CG, coffee grounds; SMC, spent mushroom compost; DDGS, distiller's dried grains with solubles; SE, standard error of mean; T × D, type × dose.

^†^ Significant (*P* < 0.05) linear (L) or quadratic (Q) contrasts of the response to incremental doses (from 0 to 300 g kg^−1^) of each by‐product.

### Production of methane

As evident from Table 6, all of the by‐products with 10, 20 and 300 g kg^−1^ DM doses significantly decreased the methane production compared with the control group (*P* < 0.05). For methane production/DDM, significant differences were observed only at the dose of 100 g kg^−1^ DM, where RAP, GAP and HC were significantly lower than the other groups.

**Table 6:** CH_4_ formation and its production per unit of dDM supply after 24 h *in vitro* incubation of the experimental diets with different inclusion levels of various by‐products using strained ruminal fluid

		Treatments				*P*					
Item	Control	10%	20%	30%	SE	Dose	L	Q	10%	20%	30%
CH_4_ (mL)						—	—	—	0.003	0.026	0.013
RAP	25.7^a^	19.0^b^	19.5^b^	18.3^b^	1.08	0.001	0.001	0.051	—	—	—
GAP	25.7^a^	17.2^b^	18.0^b^	20.0^b^	1.15	0.001	0.001	0.001	—	—	—
HC	25.7^a^	20.2^b^	17.0^b^	17.0^b^	0.88	<0.001	<0.001	0.010	—	—	—
CH	25.7^a^	22.7^ab^	20.0^bc^	17.7^c^	0.93	0.001	<0.001	0.772	—	—	—
CG	25.7^a^	20.4^ab^	20.2^ab^	17.9^b^	1.15	0.055	0.014	0.47	—	—	—
SMC	25.7^a^	19.6^b^	20.2^ab^	18.6^b^	1.05	0.045	0.021	0.253	—	—	—
DDGS	25.7^a^	24.6^a^	20.8^ab^	18.3^b^	1.16	0.050	0.002	0.699	—	—	—
CH_4_/dDM (mL kg^−1^)						—	—	—	0.005	0.103	0.263
RAP	79.3^a^	57.6^b^	57.6^b^	56.4^b^	3.47	0.001	0.001	0.027	—	—	—
GAP	79.3^a^	53.4^b^	57.2^b^	69.7^ab^	4.74	0.017	0.345	0.004	—	—	—
HC	79.3^a^	65.5^b^	70.4^ab^	75.7^ab^	2.12	0.043	0.709	0.011	—	—	—
CH	79.3	76.8	71.2	67.7	3.09	0.357	0.080	0.927	—	—	—
CG	79.3	69.9	71.4	74.9	3.94	0.808	0.736	0.410	—	—	—
SMC	79.3	65.6	69.2	68.3	3.27	0.459	0.332	0.352	—	—	—
DDGS	79.3	79.2	66.8	59.2	3.75	0.055	0.008	0.516	—	—	—

^a, b, c^ Significant differences between dietary inclusion levels are indicated with different superscript letters (*P* < 0.05).

RAP, red apple pomace; GAP, green apple pomace; HP, hempseed cake; CH, coffee hull; CG, coffee grounds; SMC, spent mushroom compost; DDGS, distiller's dried grains with solubles; dDM, digested dry matter; SE, standard error of mean; L, linear; Q, quadratic; 10%, control and 10% inclusion of by‐products; 20%, control and 20% inclusion of by‐products; 30%, control and 30% inclusion of by‐products.

For the three doses of by‐products, CH_4_ production linearly decreased by 26.0%, 24.4% and 29.0% for RAP diets (*P* < 0.05); quadratically decreased by 33.1%, 29.9% and 22.2% for GAP diets (*P* < 0.05); linearly decreased by 21.4%, 33.6% and 33.6% for HC diets (*P* < 0.05); and quadratically or linearly decreased when CH, CG, SMC and DDGS partially replaced diets at a certain dose. CH_4_/DDM linearly decreased by 27.3%, 27.4% and 28.8% for RAP diets (*P <* 0.05). CH_4_/DDM linearly decreased by 32.7% and 27.9% for GAP diets (*P* < 0.05). CH_4_/DDM linearly decreased by 25.3% for HC dose of 100 g kg^−1^ DM (*P* < 0.05).

## DISCUSSION

The increasing focus on sustainable livestock production has spurred interest in identifying alternative feed ingredients that can reduce environmental impact while maintaining or improving production efficiency. Agro‐industrial by‐products represent a promising solution due to their abundance, cost‐effectiveness and potential to enhance rumen fermentation and reduce greenhouse gas emissions. This study investigated the potential of seven distinct by‐products to enhance rumen fermentation and reduce methane emissions, offering a novel perspective by comprehensively comparing their dose‐dependent effects. The selected by‐products, including high‐protein and high‐fibre by‐products, were chosen for their nutritional diversity, availability and potential bioactive properties. The commercial applicability lies in the ability of these by‐products to provide cost‐effective, sustainable feed options while repurposing agricultural waste and mitigating methane emissions, thus supporting both economic and environmental goals.

### Nutrient profiles of tested agro‐industrial by‐products

As these potential feed ingredients are derived from agro‐industrial industries, the nutrients are basically of high content. HC, with its high crude protein content and rich rumen undegraded protein,^28^ showed potential to replace soybean meal in livestock diets. This aligns with other studies highlighting HC's high protein value and its potential to reduce reliance on imported soybean meal.^29^ Apple pomace (RAP and GAP) contained high NFC, consistent with other studies which highlight its suitability as a rapid energy source in ruminant diets.^30^, ^31^ The nutritive value of coffee by‐products was similar to that of grass silage, while the fat content (15 g kg^−1^ DM) is in accordance with previously reported values.^32^ Our study found that the ash and NFC content differed from previous reports, likely because of soil contamination. Earlier research supports this, showing lower ash‐to‐NFC ratios when SMC is processed differently.^33^, ^34^

### Effects of agro‐industrial by‐products on ruminal fermentation

The pH values recorded in our study align with the normal physiological range (5.8–6.5) reported for the rumen.^35^ This indicates that the buffered rumen fluid used successfully simulated the *in vivo* ruminal environment, ensuring that the experimental conditions closely reflected the natural ruminal conditions. It is well documented that NFC, including starch, contributes to the accumulation of VFA and lactic acid.^36^ Similar to our results, Carlos *et al*. reported that wine lees cause an increase in pH at a dose of 180 g kg^−1^ DM,^37^ but not 60 and 120 g kg^−1^ DM, possibly in relation to the NFC values.

A linear reduction in digestibility was expected with increasing doses of high‐NDF by‐products, as NDF negatively influences digestibility.^38^ The higher concentrations of NDF, ADF and lignin in these fibrous by‐products contribute to the observed decrease in DM digestibility.^39^, ^40^ This linear trend aligns with another study where digestibility of SMC diets decreased 6.2% at 0–140 g kg^−1^ DM and 15.8% at 0–300 g kg^−1^ DM, possibly due to higher unfermentable ash or lower inclusion levels.^41^ In contrast, the digestibility of apple by‐products was less affected by increasing doses but should not exceed 200 g kg^−1^ DM, as previously noted.^42^ GP was utilized to assess the fermentation of substrates, serving as an indicator of the digestibility of tested feedstuff,^43^, ^44^ strongly correlated with IVDMD. Its reduction with increasing doses of fibre‐rich by‐products aligns with previous studies.^12^

NH_3_‐N is substantially crucial for ruminal nitrogen metabolism as it is the intermediate product of protein degradation. For HC and DDGS, the high CP content could be responsible for the linear increase in ammonia concentration.^45^ In contrast to the results in our study, Antonio *et al*. reported that three by‐products at a dose of 200 g kg^−1^ DM decreased the NH_3_‐N concentrations compared to control.^46^ One possible explanation would be the negative energy–nitrogen balance in by‐product diets from their study, where excess nitrogen could not be utilized due to a lack of corresponding metabolizable energy.^47^ An exception to the quadratic trends caused by increasing doses of apple by‐products was observed, where doses of 200 and 300 g kg^−1^ DM resulted in a reduction in ammonia concentration. This aligns with the findings of a previous *in vivo* study,^48^ where dietary inclusion levels of 5, 10 and 200 g kg^−1^ DM reduced ammonia concentration by up to 32.8%, which might be explained with the low CP content in apple by‐products.^49^ Another reason might be the presence of bioactive compounds able to contribute to lower ruminal protein degradation due to the complexation of tannins and protein.^48^ On the other hand, low ruminal ammonia concentrations are linked to reduced nitrogen excretion, leading to decreased nitrogen emissions from livestock. This comparative analysis is an indication of the potential contribution of apple by‐products to a sustainable livestock production.

Furthermore, VFA are the primary products of carbohydrate fermentation in the rumen and serve as the main energy source for ruminants. Structural carbohydrates mainly produce acetate, while non‐structural carbohydrates lead to higher propionate production.^50^ The total VFA concentrations found in our study were similar among the treatments, at doses of 250 and 500 g kg^−1^ DM,^51^ in accordance with previous studies conducted in cattle and sheep,^52^, ^53^ except for CG and SMC, that could be partly explained by the fewer unfermentable carbohydrates.^54^, ^55^ Depending on nutrient profiles of diets (fibre, fat, NFC and starch), A:P reflects the rumen fermentation pattern, which is positively correlated to forage.^56^, ^57^ Propionate‐type fermentation caused by RAP, GAP and DDGS is likely to implicate a promising shift that improves dietary energy utilization efficiency as propionate is the substrate of gluconeogenesis.^58^, ^59^ Sanz *et al*. observed that replacing soybean meal and barley with pea (from 0 to 1000 g kg^−1^ DM) led to a linear decline in acetate production, an increase in butyrate and no significant changes in propionate concentration and A:P.^60^ In contrast, our study found that the acetate‐type fermentation caused by SMC might be attributed to its fat content, which can increase the propionate‐to‐acetate ratio.^56^

Incorporating agro‐industrial by‐products into livestock diets can help reduce enteric CH₄ emissions due to their chemical composition and bioactive compounds. The methane reduction observed for the by‐products studied was higher compared to previous similar studies,^61^, ^62^ which might be due to their higher fat and phenolic content, as every 10 g kg^−1^ increase in fat content reduces CH_4_ production by up to 3.8%.^63^ In addition, the bioactive compounds (i.e. polyphenols) present in the industrial by‐products function by altering the microbial community and fermentation in the rumen, thereby reducing methanogenesis.^64^, ^65^ The total phenolic content in the tested by‐products was greater compared to previous studies: 0.56–2.96 GAE g^−1^ DM for most fruits and 1.02–1.48 GAE g^−1^ DM for coffee and other spent waste by‐products.^66^, ^67^ Comparable CH₄ reduction rate was observed for grape pomace at 21.3%,^68^ likely attributable to similar levels of polyphenolic compounds present in these by‐products. Methanogenesis in rumen is primarily driven by methanogens, which utilize H₂ and CO₂ as substrates to produce methane.^69^ During the ruminal fermentation of carbohydrates, acetate and hydrogen are the products of the same biochemical reaction, so higher A:P is linked to increased methane production.^70^ Consistent with the results of A:P, high doses (200, 300 g kg^−1^ DM) of RAP, GAP, HC and DDGs presented methane‐mitigating effects. CH₄ reductions were not observed with CH, SG and SMC inclusion (mL/digested DM), likely due to lower digestibility, as indicated by the A:P results. This is in line with Mounir *et al*.,^71^ who reported that increasing the dose of SCG from 0 to 200 g kg^−1^ DM did not affect CH_4_ production per kilogram of digested organic matter, even though the polyphenol content was over 10 GAE kg^−1^ DM. The methane‐mitigating effects of GAP, RAP and HC remained significant, highlighting their substantial potential for enhancing dietary energy utilization efficiency.^58^ Intriguingly, it was previously reported that brewer's waste can reduce CH₄ production linearly by 11.1%, 27.2% and 37.0% at inclusion levels of 20, 40 and 60 g kg^−1^ DM,^72^ respectively. The greater reduction at lower inclusion levels may be due to differences in diet ingredients or bioactive compounds in the by‐products.

This study utilized a single source for each by‐product to represent its typical compositional profile, reflecting its practical use in livestock diets. While this approach facilitated controlled comparisons and dose–response analysis, it inherently limits the generalizability of the findings to other sources of the same by‐products. Variability in composition due to differences in processing, storage or geographical origin may lead to variations in fermentation outcomes. Future research should focus on evaluating multiple sources of each by‐product to account for such variability and enhance the robustness of the conclusions. Nonetheless, the observed dose–response trends provide valuable insights into the potential of these by‐products for inclusion in ruminant diets.

## CONCLUSION

Based on key fermentation parameters as critical performance indicators, the similar effects observed across by‐products at a 100 g kg^−1^ DM inclusion level suggest it as an optimal choice for practical application. In contrast, the significant differences in fermentation parameters seen with CH, CG and SMC highlight the relatively superior performance of RAP, GAP, HC and DDGS. Therefore, apple by‐products, HC and DDGS hold great potential for influencing rumen fermentation and reducing methane emissions in agro‐industry applications. The use of these by‐products not only supports sustainable farming practices but also aligns with circular economy principles, helping to reduce waste and improve resource efficiency, contributing significantly to environmental sustainability.

## CONFLICT OF INTEREST

The authors declare that they have no conflicts of interest.

## Data Availability

Data sharing not applicable to this article as no datasets were generated or analysed during the current study.
